# Cumulus Cell and Oocyte Gene Expression in Prepubertal Gilts and Sows Identifies Cumulus Cells as a Prime Informative Parameter of Oocyte Quality

**DOI:** 10.3390/biology12121484

**Published:** 2023-12-03

**Authors:** Linda Marijke Haug, Robert C. Wilson, Ann Helen Gaustad, Reina Jochems, Elisabeth Kommisrud, Eli Grindflek, Anne Hege Alm-Kristiansen

**Affiliations:** 1 Department of Biotechnology, Inland Norway University of Applied Sciences, 2318 Hamar, Norway; linda.haug@inn.no (L.M.H.); robert.wilson@inn.no (R.C.W.); reina.jochems@norsvin.no (R.J.); elisabeth.kommisrud@inn.no (E.K.); 2 Norsvin SA, 2317 Hamar, Norway; ann-helen.gaustad@norsvin.no (A.H.G.); eli.grindflek@norsvin.no (E.G.)

**Keywords:** cumulus–oocyte, metabolism, in vitro maturation, porcine, fatty acid oxidation, oxidative stress, L-carnitine

## Abstract

**Simple Summary:**

Progress has been made in pig in vitro embryo production (IVEP) over the years, but some of the remaining challenges are low embryo production rates compared to many other species. Oocyte quality is an important factor influencing IVEP. Cumulus cells (CCs) surround and support the oocyte during growth and maturation, but few studies have examined gene expression in pig CCs. This study aimed to identify novel markers of oocyte quality by examining gene expression in oocytes and CCs employing the model of oocytes collected from prepubertal animals being of lower quality than those from adult animals. This knowledge could be applied in genetic selection for oocyte quality and improve IVEP procedures, quality and success rates, particularly when using oocytes from gilts, which would be valuable in increasing genetic gain through their use in embryo transfer. New markers for oocyte quality were identified, with functions related to redox potential, L-carnitine biosynthesis, and fatty acid and glucose metabolism, where transcript abundance in CCs appeared to be more reliable parameters of oocyte quality than levels in the oocyte. A potential approach for increasing oocyte competence might be prolonging the period of support from CCs.

**Abstract:**

Cumulus cells (CCs) are pivotal during oocyte development. This study aimed to identify novel marker genes for porcine oocyte quality by examining the expression of selected genes in CCs and oocytes, employing the model of oocytes from prepubertal animals being of reduced quality compared to those from adult animals. Total RNA was extracted either directly after follicle aspiration or after in vitro maturation, followed by RT-qPCR. Immature gilt CCs accumulated *BBOX1* transcripts, involved in L-carnitine biosynthesis, to a 14.8-fold higher level (*p* < 0.05) relative to sows, while for *CPT2*, participating in fatty acid oxidation, the level was 0.48 (*p* < 0.05). While showing no differences between gilt and sow CCs after maturation, *CPT2* and *BBOX1* levels in oocytes were higher in gilts at both time points. The apparent delayed lipid metabolism and reduced accumulation of *ALDOA* and *G6PD* transcripts in gilt CCs after maturation, implying downregulation of glycolysis and the pentose phosphate pathway, suggest gilt cumulus–oocyte complexes have inadequate ATP stores and oxidative stress balance compared to sows at the end of maturation. Reduced expression of *BBOX1* and higher expression of *CPT2* in CCs before maturation and higher expression of *G6PD* and *ALDOA* after maturation are new potential markers of oocyte quality.

## 1. Introduction

Oocyte quality refers to an oocyte’s potential for successful fertilization, implantation and ultimately developing into a healthy offspring [1] and is possibly the predominant factor influencing in vitro embryo production (IVEP) [2]. During oocyte growth, which is continuous until the follicle reaches a diameter of 2–3 mm, the oocyte progressively acquires developmental competence by synthesizing and storing molecules that are necessary for sustaining meiotic maturation, fertilization and early embryo development [1,3,4,5,6].

Cumulus cells (CCs) are granulosa cells that surround the oocyte and play pivotal roles during oocyte growth and maturation [7]. Transzonal projections from the CCs penetrate the zona pellucida and are in direct contact with the oocyte cell membrane where gap junctions at the end of the transzonal projections allow for bidirectional paracrine signaling and the transfer of small molecules [8,9,10]. In this way, CCs support the oocyte in, e.g., metabolic processes and the capacity to regulate oxidative stress [9,11].

Large amounts of energy are required for the oocyte to progress through maturation and fertilization [12]. Glucose metabolism is crucial for oocyte maturation and early embryo development [9,11,12], but immature and growing oocytes have a very low capacity to metabolize glucose. Thus, this is mainly performed by CCs [9,11], with subsequent transfer of both glycolysis and the pentose phosphate pathway (PPP) metabolites to the oocyte [12]. Previous studies have demonstrated that cumulus–oocyte complexes (COCs) from prepubertal gilts showed lower levels of both transcripts and proteins [9,13] involved in glycolysis compared to COCs from sows. In addition, in vitro-produced porcine oocytes exhibit reduced glucose metabolism compared to those derived in vivo [9]. Hence, the capacity of the oocyte to metabolize glucose is suggested as a marker of oocyte developmental competence [9,13]. This relationship has, however, not been investigated separately in porcine CCs. The PPP is possibly more crucial for oocytes to acquire developmental competence than glycolysis, as the PPP, in addition to supplying energy, also plays a vital role in the prevention of cellular damage from reactive oxygen species and produces ribose 5-phosphate for purine synthesis [2,9,12]. For glucose metabolism, glucose-6-phosphate dehydrogenase (*G6PD*), encoding a rate-limiting enzyme for the PPP, and transcripts encoding two key enzymes in glycolysis, namely the platelet isoform of phosphofructokinase (PFKP) and fructose-bisphosphate A (ALDOA), could be potential biomarkers for oocyte quality. 

Porcine oocytes contain a larger total volume of cytoplasmic lipid droplets compared to other species [12,14,15]. Mature porcine oocytes contain less triglycerides, the main lipid droplet components, compared to immature oocytes, and it has been demonstrated that inhibition of lipid metabolism during in vitro maturation (IVM) resulted in decreased developmental potential [16]. The intracellular lipid store has therefore been proposed to be of special importance for adenosine triphosphate (ATP) production during porcine oocyte maturation [11,17,18,19]. Increased lipid metabolism for ATP production could, in addition, make glucose accessible for other functions than energy production, such as the PPP [17]. Long-chain fatty acids are dependent on the molecule L-carnitine for entry into the mitochondria where they are metabolized through β-oxidation before entering the tricarboxylic acid (TCA) cycle for further ATP production [20]. L-carnitine is in addition an antioxidant [20,21,22] and is found in both follicular [23] and oviduct fluids [24]. As L-carnitine is not a standard inclusion in IVM medium, it would be of interest to analyze the expression of trimethyllysine hydroxylase, epsilon (*TMLHE*), and gamma-butyrobetaine hydroxylase 1 (*BBOX1*), encoding the enzymes catalyzing the first and last step of the L-carnitine biosynthesis pathway [25], respectively, to investigate whether porcine COCs can modulate L-carnitine biosynthesis; to our knowledge, this has not yet been studied. In conjunction with the expression of carnitine palmitoyltransferase 2 (*CPT2*), an indicator of the rate of fatty acid oxidation (FAO) [26], this could contribute valuable knowledge about regulatory mechanisms of lipid metabolism in COCs and its relation to oocyte quality.

Apoptosis has previously been suggested as a marker of oocyte developmental potential. However, studies have concluded that apoptosis in oocytes is not a reliable measure of the oocyte’s quality [27,28,29], while apoptosis in CCs before maturation is proposed to be an indicator of oocyte developmental competence both in humans [30] and cattle [29]. Hussein et al. [31] demonstrated that bovine oocytes could affect apoptosis in CCs by modulating the expression of apoptosis-related genes; thus, transcript levels of pro-apoptotic bcl2 associated x (*BAX*) and anti-apoptotic bcl2 like 1 (*BCL2L1*) in CCs before and after in vitro maturation could serve as possible indicators of oocyte quality. 

Furthermore, higher expression of epidermal growth factor receptor (*EGFR*) [32,33], growth hormone receptor (*GHR*) [33] and prostaglandin-endoperoxide synthase 2 (*PTGS2*) [32,34] in CCs has led to these three genes being proposed as markers of competence in bovine and human oocytes. These gene products act through, and influence, CC function, oocyte maturation and developmental competence. Hence, their transcript abundances could be potential markers of oocyte quality in porcine CCs. 

Besides genetic factors, the intrinsic developmental potential of oocytes is primarily determined by the follicle environment [4,14,35], and oocytes collected from prepubertal animals have demonstrated reduced developmental capacity compared to those from adult animals [5,36,37,38,39,40]. Based on this, the model of prepubertal and adult oocyte donors has previously been employed to study oocytes of low versus high developmental competence in, e.g., cows [41], sheep [35,42] and pigs [27,43]. 

Increased knowledge of the interplay between oocytes and CCs could provide insight for improving IVEP procedures and consequently lead to increased oocyte and embryo quality. Improving the quality and IVEP success rate of gilt oocytes would be valuable both for their use in research and in commercial embryo transfer. Gilt oocytes are more accessible from the slaughterhouse than sow oocytes, and the transfer of embryos after IVP with gilt oocytes would lead to higher genetic gain in a breeding program. Besides evaluating oocyte quality, e.g., after altered maturation protocols, new genetic markers of porcine oocyte quality could potentially identify differences in oocyte quality between individuals and be applied in genetic selection for oocyte quality. 

The aim of this study was to identify novel markers of oocyte quality. This was performed by analyzing gene expression in immature and in vitro matured CCs and oocytes collected from prepubertal gilts and sows, focusing on genes related to metabolism, apoptosis and markers of oocyte competence identified in other species.

## 2. Materials and Methods

### 2.1. Chemicals and Media

All chemicals and reagents were purchased from Sigma-Aldrich (Oslo, Norway) unless otherwise stated. Washing of COCs was performed with porcine X medium (PXM), and maturation was performed in porcine oocyte medium (POM) [44]. Minor changes were made to the POM medium, and the final composition was 108 mM NaCl, 10 mM KCl, 0.4 mM MgSO_4_·7H_2_O, 0.35 mM KH_2_PO_4_, 25 mM NaHCO_3_, 5.0 mM glucose, 0.2 mM Na-pyruvate, 2.0 mM L-glutamine, 2.0 mM Ca-(lactate)_2_·5H_2_O, 5.0 mM hypotaurine, 0.6 mM L-cysteine, 20 mL/L BME amino acids, 10.0 mL/L MEM non-essential amino acids, 0.01 mg/mL gentamicin, 4.0 mg/mL BSA, FLI (FGF2 40 ng/mL, LIF 20 ng/mL, IGF1 20 ng/mL), 10 ng/mL epidermal growth factor and 50 µM β-mercaptoethanol (Gibco, Fisher Scientific AS, Oslo, Norway). 

### 2.2. Animal Material and Ethics

Sow and gilt ovaries were collected at a commercial abattoir, originating from random herds. Material was collected from animals that were routinely slaughtered; therefore, no ethical approval was required. In Norway, swine are cared for according to internationally recognized guidelines and regulations for keeping pigs in Norway (The Animal Welfare Act, 10 July 2009, https://www.regjeringen.no/en/dokumenter/animal-welfare-act/id571188/, accessed on 12 April 2023 and Regulations for keeping pigs in Norway, 18 February 2003, https://lovdata.no/dokument/LTI/forskrift/2003-02-18-175, accessed on 12 April 2023). Ovaries were collected from May to August 2022. 

### 2.3. Experimental Design

Prepubertal gilts are pigs that have not yet produced a litter, and their ovaries have follicles of smaller size and show no signs of ovulation (no preovulatory follicles, corpora lutea or corpora albicantia) [45], in contrast to ovaries of cycling gilts which have follicles of different sizes along with corpora lutea or corpora albicantia. The prepubertal gilts were around 5 months of age. The term sows refers to older pigs that have produced at least one litter. Sow ovaries that had follicles along with corpora lutea or corpora albicantia from previous cycles were collected [45]. At the abattoir, gilts, both prepubertal and cycling, were slaughtered separately from sows, and ovaries from sows and prepubertal gilts were collected and kept apart. Immature COCs from prepubertal gilts and sows were randomly placed into the immature or in vitro maturation groups, where total RNA was extracted from CCs and oocytes separately, either directly following aspiration or after 44 h in vitro maturation, respectively. 

There were 8 experimental groups in total: 4 groups with oocytes (prepubertal gilts before and after IVM and sows before and after IVM) and 4 groups with CCs (prepubertal gilts before and after IVM and sows before and after IVM). For all groups, triplicate pools of 50–60 oocytes or their corresponding CCs were used for RNA extraction and downstream analyses. 

### 2.4. Selection of Target Genes

To select the experimental genes, a thorough literature search was conducted to pinpoint processes that previous studies had identified as having implications for oocyte quality in porcine or other species. Glucose and lipid metabolism in both oocytes and CCs and apoptosis in CCs were found to be associated with oocyte quality, as described in the introduction section. For each of the processes of interest, rate-limiting genes and/or genes where transcript abundance had been recognized in other studies to affect oocyte quality were selected. In addition, relating to lipid metabolism, two genes involved in L-carnitine biosynthesis were selected. This study did not involve supplementation of the media with L-carnitine, but it investigated whether COCs from prepubertal gilts and sows exhibit different expression levels of genes encoding enzymes involved in L-carnitine biosynthesis. Finally, expression in CCs of a selection of three genes (*EGFR*, *GHR* and *PTGS2*), which previous studies in other species had identified as potential markers of oocyte quality, was selected. Supplementary RNA-sequencing data from Bu et al. [46] and the webtool BioGPS pigatlas (http://biogps.org/pigatlas/#goto=welcome (accessed on 1 September 2022)) were used to assess the expression levels of the potential genes in oocytes. There was little information to be found on the expression levels of these genes in porcine CCs; hence, the genes were selected based on their expression in oocytes. 

### 2.5. Cumulus–Oocyte Complex Collection and In Vitro Maturation

COC collection and in vitro maturation were performed as described by Haug et al. [47]. Sow and gilt ovaries in different phases of the estrus cycle were collected and transported to the laboratory in 0.9% NaCl at 32–36 °C within 2 h of slaughter. On arrival, ovaries were washed with 0.9% NaCl supplemented with 2.5 µg/mL kanamycin and placed in a beaker in a water bath at 34–35 °C until follicle aspiration. Follicles with a diameter of 2 to 6 mm were aspirated with an 18-gauge needle and 10 mL syringe. Oocytes with several layers of compact cumulus and evenly granulated cytoplasm, as determined by microscopy, were selected. For in vitro maturation, 25–30 COCs were washed three times in PXM and once in POM medium and transferred into each well of a Nunc^®^ four-well dish containing 500 µL of pre-equilibrated POM medium. For the first 20 h, COCs were matured in POM supplemented with 0.1 mM dibutyryl-cAMP (dbcAMP) and 0.05 IU/mL porcine FSH and LH (Insight Biotechnology Ltd., Wembley, UK). Subsequently, COCs were matured for another 24 h in POM without dbcAMP and hormones. COCs were cultured for a total of 44 h at 38.8 °C in a humified atmosphere containing 6% CO_2_ in air.

### 2.6. RNA Extraction and cDNA Synthesis

To separate the CCs from the oocytes, COCs were transferred to a 1.5 mL Eppendorf tube and vortexed for 1 min. The remaining CCs were removed by pipetting, and any oocytes with CCs still attached were discarded. Oocytes were subsequently washed with 6 drops of PXM and then 3 drops of diethyl pyrocarbonate (DEPC)-treated phosphate-buffered saline (PBS) containing 0.1% polyvinyl alcohol (PVA) (dPBS/PVA) before being transferred in 2 µL dPBS/PVA to the RNA extraction solution. Total RNA was extracted using RNAGEM^TM^ Tissue PLUS (ZyGEM, Hamilton, New Zealand) and treated with DNase I (at 80 units/mL for 10 min, a minor deviation from the manufacturer’s instructions). CCs were transferred to a 1.5 mL Eppendorf tube within 0.5 mL of dPBS/PVA and centrifuged at 1000× *g* for 4 min. The supernatant was removed, and total RNA from the cell pellets was extracted as described for oocytes. RNA concentrations were measured in all samples using a Qubit fluorometer with the Qubit^TM^ RNA High Sensitivity assay kit (Invitrogen, Oslo, Norway). RNA samples were subsequently stored at −80 °C until further use.

Total RNA from each pool of oocytes (ca. 70 ng) and 100 ng RNA from each pool of CCs was reverse-transcribed into cDNA using SuperScript IV VILO Master Mix (Invitrogen, Oslo, Norway), containing both random and oligo(dT) primers, according to manufacturer’s instructions, and then diluted 1:20 with PCR-grade water. One RNA sample from each preparation was processed without reverse transcriptase (-RT) to provide a negative control for subsequent gene expression analyses. cDNA was stored at −20 °C until further use. 

### 2.7. Quantitative PCR

Gene expression was assessed by quantitative polymerase chain reaction (qPCR) using FAM-labeled TaqMan^®^ Gene Expression Assays (Applied Biosystems, Foster City, CA, USA) available on the ThermoFisher website (http://www.thermofisher.com (accessed on 1 October 2022)). Details for each gene are listed in Table 1 and Appendix A (Table A1). Each qPCR reaction mix contained 5 μL of (2×) TaqMan Fast Advanced Master Mix (Applied Biosystems catalog No. 4444556), 0.5 μL (20×) TaqMan Assay, 0.5 μL dH_2_O and 4.0 μL cDNA template (equivalent to 1 ng RNA per reaction), giving a final reaction volume of 10 μL. All PCR reactions were performed in triplicate. QPCR was run on a 7500 Fast Real-Time PCR System (Applied Biosystems) in fast cycling mode. The qPCR reaction mixture was subjected to an initial UNG incubation at 50 °C for 2 min and then enzyme activation at 95 °C for 20 s. This was followed by 45 cycles of denaturation at 95 °C for 3 s and annealing and elongation at 60 °C for 30 s. Negative controls included -RT samples to check for genomic DNA contamination, and PCR reactions consisting of the qPCR reaction mixture without an added sample cDNA template were always included to ensure the absence of nucleic acid contamination. All -RT samples and no template controls came up negative. 

A panel of reference genes, previously validated and/or used as reliable reference genes in similar studies, namely Actin beta (*ACTB*) [27,48,49,50,51], glyceraldehyde-3-phosphate dehydrogenase (*GAPDH*) [27,46,49,52], hydroxymethylbilane synthase (*HMBS* aka *PBGD*) [53,54,55], hypoxanthine phosphoribosyltransferase 1 (*HPRT1*) [56] and tyrosine 3-Monooxygenase/Tryptophan 5-Monooxygenase Activation Protein Gamma (*YWHAG*) [49] were compared using NormFinder [57], which identified *ACTB* as the most stable reference gene for both CCs and oocytes. The transcript abundance from each gene was hence normalized against the abundance of the reference transcript *ACTB*. The relative expression of each gene was calculated using the ΔCt method with efficiency correction [58]. Mean efficiency values for each primer set were calculated from the amplification profiles of individual samples with LinRegPCR software (version 2021.2; https://medischebiologie.nl/files/ (accessed on 10 December 2022)) [59,60]. Cq values obtained from LinRegPCR were employed for all calculations. To analyze the relative change in gene expression during COC in vitro maturation, transcript abundance of in vitro matured oocytes or CCs was compared to that of immature (immature as calibrator) oocytes or CCs for gilts and sows separately. To analyze the difference in gene expression between gilts and sows of the same stage of maturation, transcript abundance in gilt oocytes or CCs was compared to that of sows (sow as calibrator) for the immature and in vitro matured groups separately.

### 2.8. Statistical Analysis

Statistical analysis was performed using RStudio version 4.1.2 (1 November 2021). Gene expression ratios were log base 10 transformed and then tested for normality using the Shapiro–Wilk test. Normally distributed data were analyzed using a two-sample t-test assuming unequal variance. The results were considered statistically significant when *p* ≤ 0.05. Graphs were plotted using GraphPad Prism version 9.0 (GraphPad Software, San Diego, CA, USA).

## 3. Results

CCs displayed greater changes in gene expression, in gilts more so than sows, than oocytes, when comparing mRNA transcript abundance of selected genes in CCs and oocytes at the time of follicle aspiration and after 44 h COC in vitro maturation. 

### 3.1. Gene Expression in Porcine Cumulus Cells

**Figure 1:**
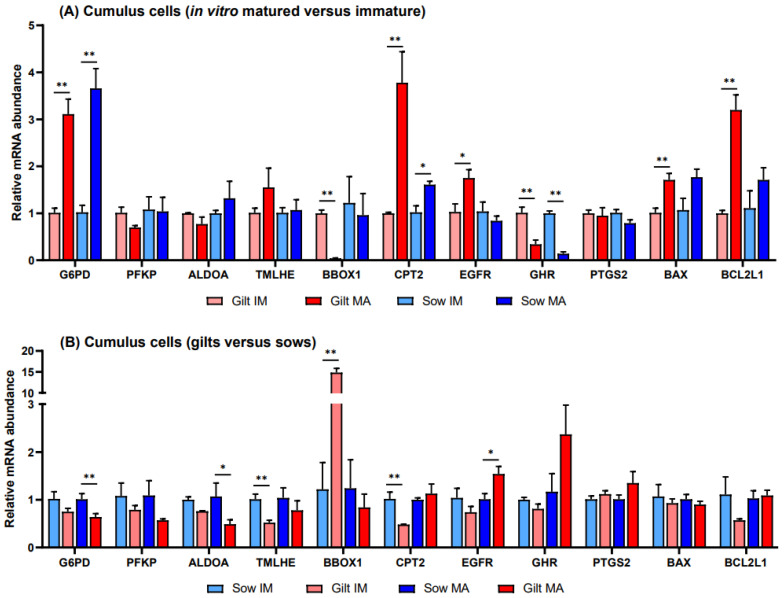
Relative transcript abundance of selected target genes in cumulus cells isolated from immature (IM) and in vitro matured (MA) cumulus oocyte complexes derived from prepubertal gilts and sows as determined by reverse transcription quantitative PCR. Data are expressed as mean ± SEM of three biological replicates per group with (**A**) in vitro matured transcript levels expressed relative to those in immature cumulus cells and (**B**) gilt transcript levels expressed relative to those in sow cumulus cells. Gene expression ratios were calculated applying the ΔCq method with efficiency correction after normalization against *ACTB*. ** indicates significant difference between bars (*p* < 0.05) while * indicates *p* < 0.10.

**Figure 2:**
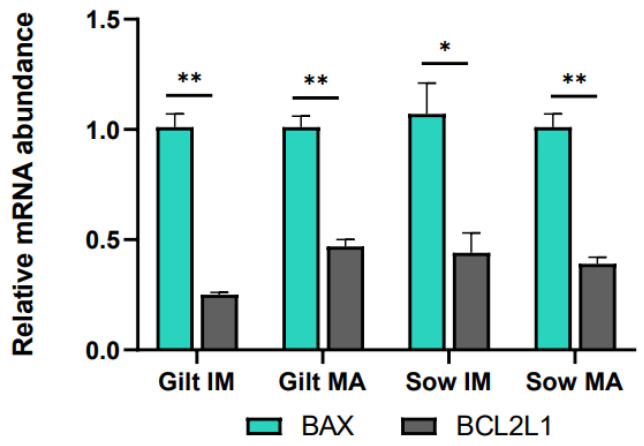
Transcript levels of *BCL2L1* relative to *BAX* in cumulus cells isolated from immature (IM) and in vitro matured (MA) cumulus–oocyte complexes derived from prepubertal gilts and sows as determined by reverse transcription quantitative PCR. Data are expressed as mean ± SEM from three biological replicates per group. Gene expression ratios were calculated by applying the ΔCq method with efficiency correction after normalization against *ACTB*. ** indicates significant difference between bars (*p* < 0.05) while * indicates *p* < 0.10.

Relative to before maturation, transcript levels of *G6PD* in CCs showed a fold increase of 3.11 (*p* = 0.002) in gilts and 3.66 (*p* = 0.002) in sows after maturation (Figure 1A). Accumulation of *CPT2* transcripts also increased for both gilts and sows, exhibiting a fold increase of 3.78 (*p* = 0.022) in gilts and 1.61 (*p* = 0.088) in sows, while *BBOX1* transcript abundance changed in gilts only, being expressed at a level of 0.04 (*p* = 0.019) post-maturation. Following maturation, *GHR* was expressed at a lower level in both gilts and sows (0.34, *p* = 0.028 and 0.14, *p* = 0.038, respectively), and there was a trend for higher expression of *EGFR* in gilts (1.75, *p* = 0.060), relative to pre-IVM. Both genes related to apoptosis were expressed at higher levels after maturation relative to before maturation in gilt CCs (Figure 1A), while *BCL2L1* displayed a higher fold increase (3.20, *p* = 0.002) than *BAX* (1.71, *p* = 0.015). However, comparing the ratio of *BCL2L1* to *BAX*, *BCL2L1* was expressed at a significantly lower level as compared to *BAX* for both gilts and sows both before and after IVM (Figure 2). 

Prior to in vitro maturation, gilt CCs showed a 14.8-fold higher level (*p* = 0.026) of *BBOX1* transcripts relative to CCs from sows, while *TMLHE* and *CPT2* exhibited levels of 0.52 (*p* = 0.010) and 0.48 (*p* = 0.037), respectively (Figure 1B). After maturation there were no significant differences between gilts and sows in the transcript levels of genes involved in FAO, while transcripts encoding key enzymes in glucose metabolism, G6PD and ALDOA, were present in gilt CCs at levels of 0.64 (*p* = 0.044) and 0.49 (*p* = 0.070), respectively, relative to those in sows. In addition, *EGFR* displayed a 1.54-fold (*p* = 0.058) elevated level after maturation in gilt CCs as compared to sows. 

### 3.2. Gene Expression in Porcine Oocytes

**Figure 3:**
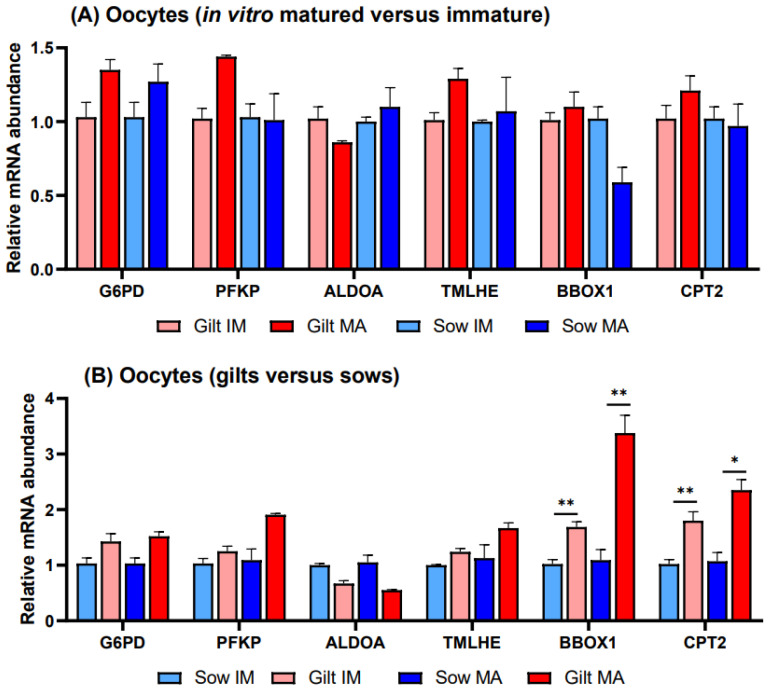
Relative transcript abundance of selected target genes in oocytes isolated from immature (IM) and in vitro matured (MA) cumulus–oocyte complexes derived from prepubertal gilts and sows as determined by reverse transcription quantitative PCR. Data are expressed as mean ± SEM of three biological replicates per group with (**A**) in vitro matured transcript levels expressed relative to those in immature oocytes and (**B**) gilt transcript levels expressed relative to those in sow oocytes. Gene expression ratios were calculated by applying the ΔCq method with efficiency correction after normalization against *ACTB*. ** indicates significant difference between bars (*p* < 0.05) while * indicates *p* < 0.10.

There were no significant differences in transcript abundance when comparing in vitro matured vs. immature oocytes derived from gilts or sows (Figure 3A). Investigating gene expression in gilts relative to sows (Figure 3B), transcripts of two genes related to FAO exhibited higher relative transcript abundance in gilt oocytes, with *BBOX1* showing a 1.69-fold (*p* = 0.027) level before and a 3.38-fold (*p* = 0.036) elevated level after maturation and *CPT2* showing a 1.80-fold (*p* = 0.044) level before and a 2.35-fold (*p* = 0.058) higher relative level after maturation. None of the differences observed in gene transcripts related to glucose metabolism were significant. 

### 3.3. The Rate of the Pentose Phosphate Pathway Relative to Glycolysis

**Figure 4:**
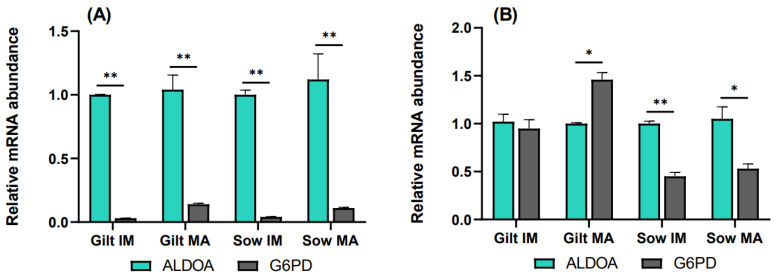
Transcript levels of *G6PD* relative to *ALDOA* in (**A**) cumulus cells and (**B**) oocytes isolated from immature (IM) and in vitro matured (MA) cumulus–oocyte complexes derived from prepubertal gilts and sows as determined by reverse transcription quantitative PCR. Data are expressed as mean ± SEM from three biological replicates per group. Gene expression ratios were calculated by applying the ΔCq method with efficiency correction after normalization against *ACTB*. ** indicates significant difference between bars (*p* < 0.05) while * indicates *p* < 0.10.

To elucidate the relative rate of the PPP compared to glycolysis, transcript levels of *G6PD* were analyzed relative to *ALDOA*. In cumulus cells, *G6PD* transcripts accumulated to a significantly lower level relative to *ALDOA* for all the experimental groups, as shown in Figure 4A. In gilt oocytes (Figure 4B), transcript abundance of *G6PD* was nearly equivalent to that of *ALDOA* prior to maturation, while it was 1.46-fold (*p* = 0.06) higher compared to *ALDOA* after maturation. Sow oocytes demonstrated the opposite relationship, where *G6PD* was expressed at lower levels both before (0.45, *p* = 0.036) and after (0.53, *p* = 0.087) maturation, relative to *ALDOA*.

## 4. Discussion

In this study, the expression of genes involved in glucose and lipid metabolism was evaluated in both oocytes and CCs from gilts and sows. In addition, the expression of genes associated with apoptosis and oocyte quality in other species was evaluated in CCs. 

### 4.1. Glucose Metabolism

Oocytes stimulate glycolysis in CCs, but fully grown oocytes will promote higher levels of glycolysis than those not having completed the growth phase [61]. In the current study, reduced transcript levels of *ALDOA* in gilt CCs after maturation indicate glycolysis was downregulated in gilt CCs as compared to sows, suggesting gilt oocytes had not finished the growth phase. Hence, higher expression of *ALDOA* in CCs after maturation is a potential marker of oocyte quality. Although the same pattern of transcript abundance for *ALDOA* was observed in oocytes, the differences were not significant. It might be that more significant differences would have been detected if a higher number of biological replicates had been included. 

In vitro cultured oocytes and embryos are usually exposed to higher levels of oxidative stress than those derived in vivo [15,61,62]. The PPP supports cellular redox balance by producing NADPH, which is involved, e.g., in converting oxidized glutathione into reduced glutathione, an antioxidant critical for the developmental potential of oocytes [9,63]. NADPH can be transferred from CCs to oocytes via gap junctions, and for both gilt and sow CCs, *G6PD* levels indicated significant upregulation of the PPP after maturation, supporting a vital role for the PPP in COC maturation [2,17,64]. The *G6PD* transcript abundance suggested, however, at the same time, downregulation of the PPP in gilt compared to sow CCs, which suggests that gilt COCs might be less competent in regulating oxidative stress.

Elevated expression of *G6PD* in more competent porcine oocytes has been reported [65], but this was not observed in the current study, nor by Yuan et al. [52]. Although no significant difference in *G6PD* expression was observed between gilt and sow oocytes, the total COC contribution towards supporting the redox balance through the PPP was reduced in gilt COCs. Abundant expression of *G6PD* might be a more reliable marker of oocyte quality when measured in CCs than in oocytes, but this would need further investigation.

To compare the rate of the PPP to glycolysis, *ALDOA* was preferred over *PFKP* as a measure of the rate of glycolysis as more significant differences were detected between the groups for *ALDOA*, and therefore *ALDOA* did, in this study, appear more rate-limiting for glycolysis than *PFKP*. The results suggest a stronger relative contribution from the PPP in porcine oocytes than in CCs, which was also observed in bovine COCs [9,62]. The main contribution of CCs in glucose metabolism is through supplying oocytes with pyruvate and lactate, further validating the use of elevated expression of *ALDOA* in CCs as a marker of oocyte quality. Further metabolism of pyruvate will cause increased production of reactive oxygen species [14,66,67], and it could be speculated whether high rates of glycolysis in fully grown oocytes are regulated by their redox state, as only fully grown COCs can sustain a higher oxidative stress response.

### 4.2. Fatty Acid Oxidation

The amount of ATP in oocytes has been linked to its further developmental potential [17,18,19,23,68]. Since glycolysis appeared to be downregulated in gilt CCs in this study, gilt COCs would have to rely more on lipid metabolism as a supply of energy. In accordance with this, gilt oocytes displayed a higher transcript abundance of genes involved in FAO as compared to sow oocytes, which corroborates previous studies on porcine oocytes [27,52]. As discussed in a study on oocytes from prepubertal and cycling gilts [69] and bovine oocytes of contrasting quality [70], this most likely reflects prepubertal gilt oocytes not having finished the growth phase and therefore needing a higher expression of the transcripts encoding enzymes involved in ATP synthesis. In contrast, sow oocytes have completed the necessary accumulation of ATP and show a lower more stable expression of these transcripts. In gilt CCs, transcript abundance of *CPT2* suggested FAO was significantly downregulated before maturation compared to sows, but no difference in *CPT2* was detected after maturation. This apparent delay in FAO in both gilt oocytes and CCs, together with the lower rate of glycolysis in CCs, implies gilt oocytes might not have adequate stores of ATP at the end of IVM. Studies of amino acid metabolism could provide additional information and would be of interest to include in the future. Excessive fatty acid levels in the follicular fluid or medium can also cause increased FAO in CCs to protect oocytes from lipotoxicity [71]. But seeing a similar pattern in both CCs and oocytes before and after maturation suggests the FAO in CCs observed in this study rather reflects the need for ATP in the COCs.

CC transcript abundance needs to be interpreted differently from that in oocytes, as transcript levels in CCs reflect protein synthesis demands in COCs, while those in oocytes also reflect their stage of development and maturation. When oocytes have completed the growth phase, transcription ceases [72,73]. From meiotic resumption and until embryonic genome activation, oocyte and embryonic development relies on post-transcriptional regulation for modulating protein synthesis [42,72]. Consequently, after transcription is silenced, there is a gradual decline in transcript abundance observed during oocyte maturation [74,75,76]. In porcine IVM, the standard is to include dbcAMP in the medium for the first 20 h to prevent meiotic resumption, followed by 24 h without dbcAMP supplementation [44,77]. Hence, if the oocyte was not fully grown at aspiration, transcription could continue during the first 20 h of IVM. This might further explain the differences observed between gilt and sow oocytes in transcript abundance of, e.g., *CPT2*, as gilt oocytes probably still had active transcription in the first part of IVM, while the fully grown sow oocytes would have minimal transcription and rely on the stored supply of RNA. In hindsight, analyzing transcript abundance also after 20 h maturation would have added valuable information on transcriptional activity in the space of time between meiotic resumption and the end of maturation.

### 4.3. L-Carnitine Biosynthesis

The most striking result in this study is perhaps the interplay between the L-carnitine biosynthesis pathway gene *BBOX1* and *CPT2*, a measure of the rate of FAO. This is most clearly demonstrated in the CCs where the level of *CPT2* transcripts suggests that FAO was downregulated in gilt CCs at the time of aspiration. We hypothesize this was caused at least in part by an insufficiency in L-carnitine as indicated by elevated levels of *BBOX1* transcripts, as a proxy for enhanced BBOX1 protein amounts. Building on this hypothesis, which needs to be further validated by functional assays, after maturation, there appears to be sufficient L-carnitine to sustain a similar level of FAO in gilt CCs to that seen in sows. This hypothesized delay in L-carnitine biosynthesis, and hence the rate of FAO, appeared to be more pronounced in gilt oocytes in contrast to CCs, with a difference in *BBOX1* and *CPT2* transcript abundance still being present in gilt oocytes after maturation, relative to sows. This hypothesis is supported by the study of Godárová et al. [78], which demonstrated that insufficient levels of L-carnitine caused a reduction in *CPT2* transcript abundance, while the abundance of *CPT2* increased significantly within 48 h of L-carnitine supplementation. The results from the current study indicate that *BBOX1* encodes the more rate-limiting enzyme for L-carnitine biosynthesis than *TMLHE* in both CCs and oocytes as more significant differences in transcript abundance were observed for *BBOX1*.

In contrast to this study in pigs, Montjean et al. [23], although employing different methods, found no expression of genes encoding enzymes in L-carnitine biosynthesis in human oocytes. A correlation has been proposed between the amount of fatty acids in an oocyte and the importance of FAO for oocyte and preimplantation embryo development [14,17]. As porcine oocytes contain a very large amount of fatty acids compared to most other species, approximately 156 ng fatty acid per porcine oocyte as compared to, e.g., 4 ng per murine oocyte [14], it should perhaps not be surprising that porcine oocytes exhibit expression of *TMLHE* and *BBOX1*. RNAs can travel from CCs to oocytes through gap junctions [72,79], but the high level of *BBOX1* in oocytes after maturation, when the gap junctions are supposed to be closed [80], suggests *TMLHE* and *BBOX1* are also transcribed by the oocytes. Nevertheless, this study indicates, for the first time, that porcine COCs can differentially express the genes involved in the synthesis of L-carnitine.

The current study did not examine the effect of L-carnitine supplementation. Multiple other studies have, however, investigated the effect of supplementing either the in vitro maturation or culture medium with L-carnitine, and it has been reported to have the potential to accelerate nuclear maturation [81], increase blastocyst development [21,22] and increase embryo survival after cryopreservation [21,82]. There is, however, a lack of consensus between studies regarding both optimal concentrations and experimental outcomes, as reviewed in the work of Carrillo-González et al. [83] which found no effect of L-carnitine supplementation on embryo production or post-vitrification survival rate. These inconclusive results might be explained by the varying levels of L-carnitine synthesized by COCs.

In addition to increasing lipid metabolism, L-carnitine is an antioxidant, and there seems to be agreement on its ability to decrease the level of reactive oxygen species [21,22,81,84]. If our hypothesis concerning the delay in L-carnitine biosynthesis in gilt COCs is correct, this may affect both their rate of FAO and redox potential, and gilt COCs could possibly benefit from L-carnitine supplementation during the first part of IVM. This is in accordance with the findings of Carrillo-González et al. [83], who suggested L-carnitine supplementation would predominantly have a positive effect when applied to poor-quality oocytes. Additional L-carnitine supplementation should, however, be applied with caution as the total concentration of L-carnitine will vary depending on amounts synthesized by the COCs, and excessive concentrations have demonstrated negative effects [22].

### 4.4. Apoptosis

Apoptosis is another potential indicator of COC quality. When anti-apoptotic *BCL2L1* is expressed at a lower level than pro-apoptotic *BAX*, it could promote apoptosis [85]. This was observed both before and after maturation in both gilt and sow CCs and could possibly be explained as a reaction to oxidative stress experienced during ovary collection and follicle aspiration [29]. Pawlak et al. [69] proposed that CCs from prepubertal gilts would display less apoptosis after IVM than those from cycling gilts, as prepubertal gilt oocytes would not have completed maturation and would therefore still need their CCs for support. In this study, *BCL2L1* was expressed at a higher level in gilt CCs after IVM as compared to before IVM, albeit at a similar level to that seen in sows, and *BCL2L1* was still expressed at a lower level relative to that of *BAX*. It is difficult to draw any conclusions regarding the function of apoptosis from the current study. According to Yuan et al. [29], CC apoptosis is rather common in the periphery of COCs, and an analysis of oocyte-proximal and -distal CC populations could have potentially added insight to this study.

### 4.5. Gene Expression of PTGS2, GHR and EGFR

Although higher expression of *PTGS2* in CCs has previously been correlated to a higher oocyte developmental competence in cows [32] and humans [34], no such relationship was seen in the current study. A possible explanation could be that transcription of *PTGS2* was completed at an earlier stage of development, as suggested by Regassa et al. [13], who also failed to see a relationship between *PTGS2* expression and oocyte quality in their study of bovine COCs.

The follicular fluid contains growth hormone (GH), but it was not included in the IVM medium. Regardless, the levels of *GHR* transcripts were measured both pre- and post-IVM since an increase in abundance following maturation could indicate the COCs still required GH. Both gilt and sow CCs did, however, demonstrate reduced accumulation of *GHR* transcripts post-IVM. Even so, high variability was observed, with CCs from one biological sample from prepubertal gilts displaying increased *GHR* expression following maturation, which could indicate this specific group of COCs would have benefited from GH being included in the IVM medium. Higher variability between the biological replicates in prepubertal animals has also been reported in other studies [41]. There were no significant differences observed in *GHR* transcript abundance between gilts and sows, neither for CCs nor for oocytes, and, therefore, *GHR* expression in COCs did not seem to be a marker of oocyte quality in this study.

The increased level of *EGFR* expression observed in gilt CCs after maturation could be explained by delayed development of these receptors in less competent oocytes [80]. Consequently, gilt COCs would have responded poorly to epidermal growth factors (EGFs) while *EGFR* transcript abundance was low. It can be speculated whether *EGFR* expression was even lower compared to sows at an earlier stage of development, prior to aspiration. However, cumulus cell transzonal projections begin to withdraw from the oocyte around the time of meiotic resumption [9], but EGF signaling can prolong gap-junctional communication [86]. Therefore, the increased abundance of *EGFR* transcripts could also be a response from the COCs to reduce the withdrawal of transzonal projections while there was still a need for the exchange of factors between the gilt CCs and oocytes. Prolonging the period of functional gap junctions during IVM could potentially enhance COC quality [87]. It was not the intention of this study to characterize the mechanism of *EGFR* per se. Still, it would have been interesting to have analyzed the levels of follicle-stimulating hormone receptor (*FSHR*), as follicle-stimulating hormone (FSH) influences the activation of *EGFR* [80].

This study did not document a correlation between higher transcript abundance of *PTGS2*, *GHR* and *EGFR* and enhanced oocyte quality. It is possible that the expression of these genes needs to be measured at an earlier stage of development for their meaningful use as markers of oocyte quality in pigs. This would, however, not be practicable as it would mean analyzing oocytes from follicles of less than 2 mm diameter, when they have not yet finished the growth phase.

## 5. Conclusions

To identify novel markers of oocyte quality, the close association between CCs and oocytes makes it crucial to examine gene expression in both cell types. For several genes, transcript abundance in CCs seemed to be a more reliable parameter of oocyte quality than levels in oocytes. In addition, gene expression in CCs can potentially exhibit a greater response to environmental conditions during maturation than that in oocytes. Reduced expression of *BBOX1* and higher expression of *CPT2* in CCs before maturation and transcript abundance for both *G6PD* and *ALDOA* after maturation were found to be potential markers of oocyte quality. The results from the current study indicate growth and maturation of oocytes are delayed in gilt COCs, through the downregulation of genes encoding enzymes important for glycolysis and the PPP, and an apparent delay in FAO in both oocytes and CCs, in addition to delayed EGFR signaling in gilt CCs. Hence, gilt COCs might not have adequate stores of ATP and are less competent in responding to oxidative stress compared to sow COCs at the end of maturation. Other studies have explored supplementing media with L-carnitine to increase oocyte quality, but we hypothesize porcine oocytes and CCs have the potential to differentially regulate L-carnitine biosynthesis. Further studies are needed to verify these indications and hypotheses by measuring actual levels of ATP, oxidative stress and L-carnitine in gilt and sow oocytes.

## Figures and Tables

**Table 1:** Summary of genes analyzed by reverse transcription quantitative PCR in porcine oocytes and cumulus cells.

Gene Symbol	Function	Accession Number	TaqMan^®^ Assay
*G6PD*	Pentose phosphate pathway	XM_003360515.5 ^††^	Ss02690824_g1
*PFKP*	Glycolysis	XM_021065066.1	Ss06887532_m1
*ALDOA*	Glycolysis	XM_021087995.1 ^††^	Ss06920688_m1
*TMLHE*	Carnitine biosynthesis	XM_003135511.4 ^††^	Ss06886117_m1
*BBOX1*	Carnitine biosynthesis	XM_021083234.1 ^††^	Ss06906097_m1
*CPT2*	Fatty acid metabolism	NM_001246243.1	Ss04322743_m1
*GHR* ^†^	Growth hormone receptor	NM_214254.2 ^††^	Ss03383662_u1
*EGFR* ^†^	Epidermal growth factor receptor	NM_214007.1	Ss03393423_u1
*PTGS2* ^†^	Cumulus cell expansion and function	NM_214321.1	Ss03394694_m1
*BAX* ^†^	Pro-apoptotic	XM_003127290.5 ^††^	Ss03375842_u1
*BCL2L1* ^†^	Anti-apoptotic	NM_214285.1 ^††^	Ss03383783_s1
*ACTB*	Cytoskeletal structural protein	XM_003124280.5 ^††^	Ss03376563_uH
*GAPDH*	Glycolysis	NM_001206359.1 ^††^	Ss03375629_u1
*HMBS*	Heme biosynthesis	NM_001097412.1 ^††^	Ss03388782_g1
*HPRT1*	Recycling of purines	NM_001032376.2	Ss03388274_m1
*YWHAG*	Signal transduction	XM_005661962.3	Ss06938931_s1

^†^ These transcripts were only analyzed in cumulus cells. ^††^ TaqMan^®^ Assay targets specific transcript variants, see Appendix A (Table A1) for details.

## Data Availability

The data that support the findings of this study are available from the corresponding author upon reasonable request.

## Appendix A

**Table A1:** Complete list where the TaqMan^®^ Assay will detect specific transcript variants.

Gene Symbol	TaqMan^®^ Assay	Accession Numbers of Transcript Variants
*G6PD*	Ss02690824_g1	XM_021080744.1 (X1), XM_003360515.5 (X2)
*ALDOA*	Ss06920688_m1	XM_021087995.1 (X1), XM_021087996.1 (X2), XM_021087997.1 (X3), XM_021087998.1 (X4), XM_021088000.1 (X6), XM_021088001.1 (X7), XM_021088002.1 (X8), XM_021088004.1 (X9)
*TMLHE*	Ss06886117_m1	XM_003135511.4 (X1), XM_021080447.1 (X2), XM_021080448.1 (X3), XM_021080450.1 (X4), XM_005674022.3 (X5)
*BBOX1*	Ss06906097_m1	XM_021083234.1 (X1), XM_021083235.1 (X2), XM_021083236.1 (X3)
*GHR*	Ss03383662_u1	XM_021076567.1 (X1), XM_013990636.2 (X2), XM_021076568.1 (X3), XM_021076569.1 (X4), XM_021076570.1 (X5), XM_021076571.1 (X6), XM_021076572.1 (X7), XM_021076573.1 (X8), XM_021076574.1 (X9), XM_013990637.2 (X10), XM_021076575.1 (X11)
*BAX*	Ss03375842_u1	XM_003127290.5 (X1), XM_013998624.2 (X2)
*BCL2L1*	Ss03383783_s1	XM_021077292.1 (X1), XM_021077293.1 (X2), XM_021077294.1 (X3), XM_021077295.1 (X4), XM_021077296.1 (X5), XM_021077297.1 (X6), XM_021077298.1 (X7)
*ACTB*	Ss03376563_uH	XM_003124280.5 (X1), XM_021086047.1 (X2)
*GAPDH*	Ss03375629_u1	XM_021091114.1 (X2)
*HMBS*	Ss03388782_g1	XM_021102144.1 (X1), XM_021102146.1 (X2), XM_021102147.1 (X3), XM_021102148.1 (X4)
*HPRT1*	Ss03388274_m1	XM_021079503.1 (X1), XM_021079504.1 (X2)
